# Methadone Requires the Co-Activation of μ-Opioid and Toll-Like-4 Receptors to Produce Extracellular DNA Traps in Bone-Marrow-Derived Mast Cells

**DOI:** 10.3390/ijms25042137

**Published:** 2024-02-10

**Authors:** Frida L. Martínez-Cuevas, Silvia L. Cruz, Claudia González-Espinosa

**Affiliations:** 1 Departamento de Farmacobiología, Centro de Investigación y de Estudios Avanzados del Instituto Politécnico Nacional (Cinvestav, IPN), Unidad Sede Sur, Calzada de los Tenorios No. 235, Col. Rinconada de las Hadas, México City CP 14330, Mexico; fridamartinezcuevas@cinvestav.mx; 2 Centro de Investigación Sobre el Envejecimiento, Centro de Investigación y de Estudios Avanzados del Instituto Politécnico Nacional (Cinvestav, IPN), Unidad Sede Sur, Calzada de los Tenorios, No. 235, Col. Rinconada de las Hadas, México City CP 14330, Mexico

**Keywords:** methadone, µ-opioid receptors, TLR4 receptors, mast cells, ETosis, mast cell extracellular traps

## Abstract

Methadone is an effective and long-lasting analgesic drug that is also used in medication-assisted treatment for people with opioid use disorders. Although there is evidence that methadone activates μ-opioid and Toll-like-4 receptors (TLR-4s), its effects on distinct immune cells, including mast cells (MCs), are not well characterized. MCs express μ-opioid and Toll-like receptors (TLRs) and constitute an important cell lineage involved in allergy and effective innate immunity responses. In the present study, murine bone-marrow-derived mast cells (BMMCs) were treated with methadone to evaluate cell viability by flow cytometry, cell morphology with immunofluorescence and scanning electron microscopy, reactive oxygen species (ROS) production, and intracellular calcium concentration ([Ca^2+^]*i*) increase. We found that exposure of BMMCs to 0.5 mM or 1 mM methadone rapidly induced cell death by forming extracellular DNA traps (ETosis). Methadone-induced cell death depended on ROS formation and [Ca^2+^]*i*. Using pharmacological approaches and TLR4-defective BMMC cultures, we found that µ-opioid receptors were necessary for both methadone-induced ROS production and intracellular calcium increase. Remarkably, TLR4 receptors were also involved in methadone-induced ROS production as it did not occur in BMMCs obtained from TLR4-deficient mice. Finally, confocal microscopy images showed a significant co-localization of μ-opioid and TLR4 receptors that increased after methadone treatment. Our results suggest that methadone produces MCETosis by a mechanism requiring a novel crosstalk pathway between μ-opioid and TLR4 receptors.

## 1. Introduction

Opioids are essential analgesics utilized in chronic pain treatment [1]; despite their efficacy, their use can lead to dependence and other unwanted effects in the central nervous system (CNS) and peripheral organs [1]. Among the clinically relevant opioid analgesic drugs, methadone is a long-lasting pharmacological agent used for cancer pain [2] and treatment for people with opioid use disorders as part of medication-assisted treatment (MAT) programs [3].

In vitro studies have shown that opioids produce immunomodulatory effects by altering distinct immune parameters, such as cytokine secretion, innate immune cell activation, and T-cell phenotypes [4,5,6,7]. Methadone particularly affects the normal function of T lymphocytes, monocytes, and natural killer (NK) cells [8,9,10,11].

Opioids can also produce inflammation-associated cell death [4,5,6,7,12,13]. In contrast with other opioids, methadone causes the death of leukemic cells [14,15], making it a relevant candidate for the control of cancer cell proliferation [16]. Like other opioid receptor agonists, methadone activates classical opioid G protein-coupled receptors (GPCR) coupled to Gi/o proteins, producing inhibition of cAMP production and ion channel modulation [17]. On the other hand, methadone can also bind and activate Toll-like (TLR)-4 receptors, triggering the canonical NF-κB-mediated signaling pathways with the resultant cytokine expression [18]. Due to the extensive use of methadone, the identification of receptors involved in its therapeutic and unwanted side effects is an active research field.

Despite existing information on the actions of methadone in immune cells, studies analyzing its effects on tissue-resident cell lineages, such as mast cells (MCs), are limited. MCs are myeloid cells that rapidly orchestrate inflammatory responses against microorganisms and tissue damage. They play a central role in acute allergic reactions by releasing histamine, proteases, and proinflammatory compounds after activating the high-affinity IgE receptor (FcεRI). They also trigger innate immunity responses against pathogens through the production of distinct cytokines and lipids after activating pattern recognition receptors (PRRs), such as Toll-like receptor (TLR)-4 [19,20]. To orchestrate immune responses against pathogens, MCs can produce DNA-extracellular traps (MCETs) [21]. In such circumstances, nuclear DNA is extruded from the cells along with distinct proteins, such as citrullinated H3 histone and tryptase, leading to an extracellular-trap-related cell death called MCETosis [22,23]. MCs express functional opioid receptors coupled to the activation of MAPK [24]. Remarkably, distinct crosstalk events between opioid and TLR4 receptors have been reported in that cell type, mainly associated with the opioid-dependent inhibition of the TLR4-signaling cascade [25].

This study aimed to characterize the effects of methadone in bone-marrow-derived mast cells (BMMCs) together with the receptors and signaling pathways involved. We tested the hypothesis that methadone modifies the physiology of MCs with the participation of µ-opioid and TLR4 receptors. Our results show that (a) methadone causes BMMCs’ death by inducing MCETosis, (b) ROS production and intracellular Ca^2+^ increase are essential for this process to occur, and (c) µ-opioid and TLR4 receptors closely interact to promote the formation of extracellular DNA traps (ET).

## 2. Results

### 2.1. Methadone Induces Cell Death in BMMCs in A Concentration-Dependent Manner

**Figure 1:**
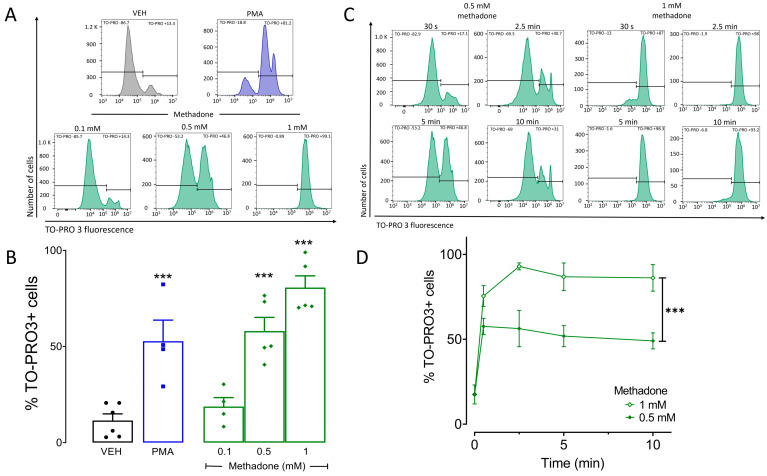
Methadone induces rapid death of BMMCs in a concentration-dependent manner. Five-hundred thousand BMMCs were exposed to different methadone concentrations. Cell viability was assessed by flow cytometry using TO-PRO3. (**A**) Representative histograms showing TO-PRO3-positive cells in control BMMCs treated with vehicle (VEH) and cells exposed to 1 μM PMA for 4 h or to methadone (0.1, 0.5, or 1 mM) for 10 min. (**B**) Quantification of methadone-induced TO-PRO3-positive cells in the experiments described in A; *** *p*< 0.001; Dunnett’s test. (**C**) Histograms of representative experiments depicting the time course of BMMC death caused by 0.5 mM or 1 mM methadone. (**D**) Quantification of TO-PRO3-positive cells in response to 0.5 mM or 1 mM methadone over time; *** *p* < 0.001, two-way ANOVA for repeated measures. Horizontal lines in (**A**,**C**) delimit the fluorescence values corresponding to TO-PRO negative and positive cells.

Stimulation of µ-opioid receptors can induce the death of neurons and other cells [12,13,26,27]. For this reason, we evaluated cell viability in BMMCs incubated with methadone (0.1, 0.5, and 1 mM) for 10 min, using phorbol myristate acetate (PMA, 1 μM, for 4 h) as a positive control [28,29]. This time of PMA treatment was chosen based on time-course experiments previously conducted in our laboratory (Supplemetary Figure S1). As expected, PMA caused 52.7% of BMMCs to die (Figure 1A). Methadone did not affect cell viability at 0.1 mM, but 0.5 mM and 1 mM produced 58% and 86.6% cell mortality, respectively (F_(4,19)_ = 20.9; *p* < 0.0001, one-way ANOVA; Figure 1B). To determine the time course of cell death occurrence, we incubated BMMCs with 0.5 or 1 mM methadone for 30 s, 2.5, or 5 min and found dead cells since the first 30 s (time: F_(4,30)_ = 25.5, *p* < 0.0001; dose: F_(1,30)_ = 37.2; *p* = 0.0001; interaction: F_(4,30)_ = 3, *p* = 0.03; two-way ANOVA for repeated measures; Figure 1C,D). These results indicate that methadone induces quick and concentration-dependent death in BMMCs.

### 2.2. Methadone Induces Cell Death through the Formation of DNA Extracellular Trap (MCETosis) in BMMCs

**Figure 2:**
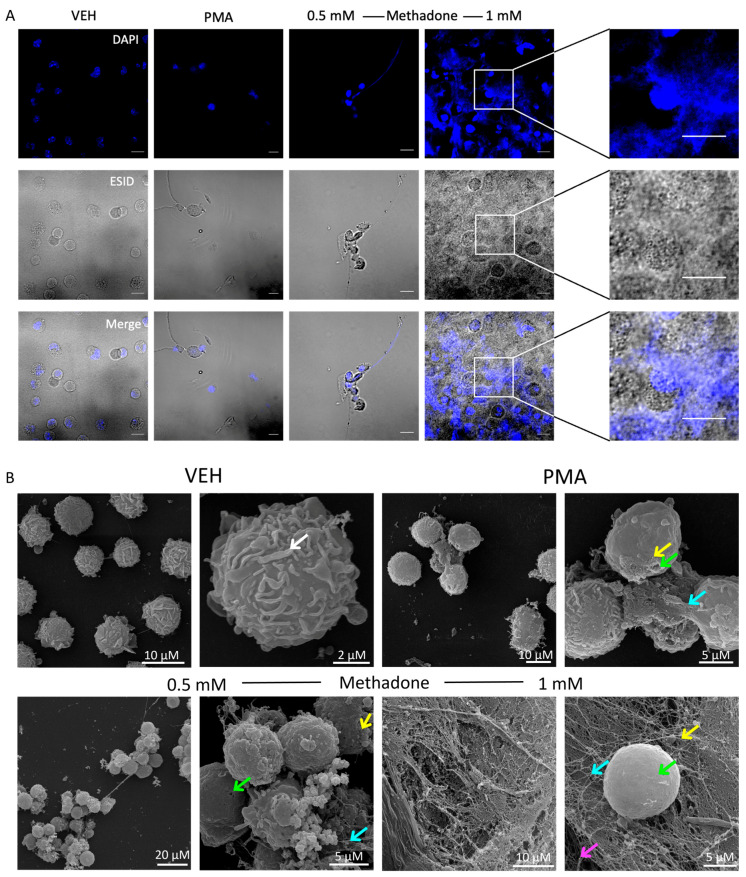
Methadone induces extracellular DNA trap formation in BMMCs. (**A**) Confocal images of control BMMCs treated with vehicle (VEH) for 10 min, 1 μM PMA for 4 h, or 0.5 mM or 1 mM methadone for 10 min. Nuclei were labeled using DAPI. A representative image from three distinct experiments performed with independent cultures of BMMCs is shown. Scale bar, 10 µm; objective 40×. (**B**) Scanning microscopy images of ET formation produced by PMA and methadone in BMMCs. The white arrow indicates membrane ruffling, and the green arrows point out discontinuities in the plasma membrane. The yellow arrow indicates small bodies, and the blue arrow shows DNA. Pink arrows indicate nucleosomes. Representative images from one of three independent experiments.

To investigate the type of cell death produced by methadone, we analyzed BMMCs treated with 0.5 mM or 1 mM for 10 min utilizing confocal microscopy and the DNA marker DAPI. Vehicle-treated cells had intact nuclei and plasmatic membranes. PMA and 0.5 mM methadone promoted the formation of cell clumps, and extracellular DNA was distinguished in some cells. At 1 mM, methadone produced DNA extrusion accompanied by nuclear and plasmatic membrane rupture, consistent with MCET formation (Figure 2A). The characteristic membrane ruffles of BMMCs (white arrow) in control cells were evident in electronic scanning microscopy images (Figure 2B). PMA induced cell clump formation (orange arrow) and DNA secretion (blue arrow). Methadone, at 0.5 mM, induced cell clustering (orange arrow), the loss of membrane ruffles, DNA ET formation (blue arrow), and granular secretion (yellow arrow), accompanied by plasma membrane disruption (green arrow). At 1 mM, methadone caused massive DNA release and extrusion of granular materials and histones (pink arrow).

### 2.3. Methadone-Induced MCETs Contain CitH3 and Tryptase

**Figure 3:**
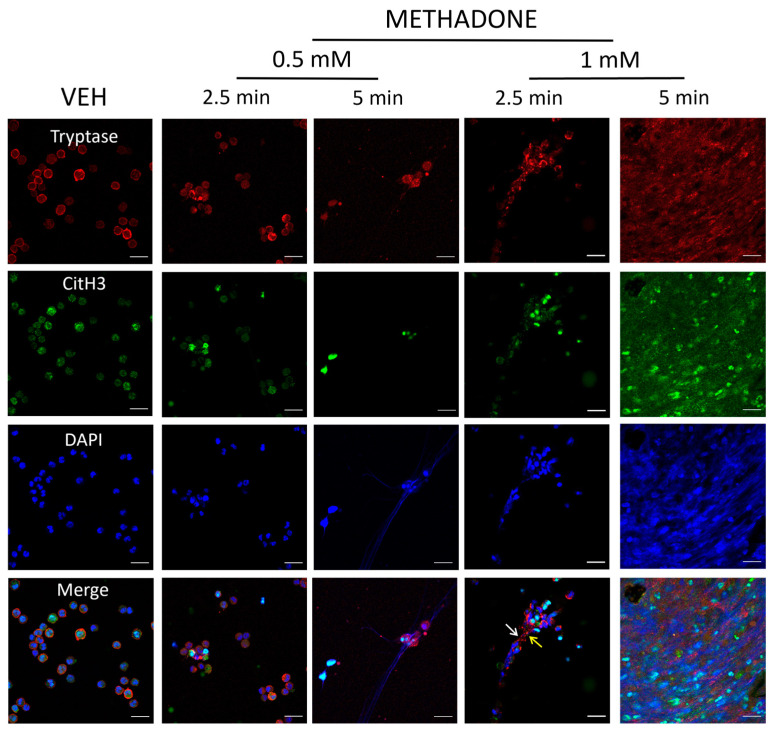
Methadone-induced MCETs contain tryptase and CitH3. Representative confocal microscopy images from three distinct experiments performed with independent BMMC cultures. Red: tryptase; green: CitH3; blue: DNA. Scale bar: 20 µm, objective: 40×. BMMCs were treated with vehicle (VEH) for 5 min or methadone (0.5 or 1 mM) for 2.5 or 5 min. The white and yellow arrows indicate tryptase and CitH3 immunoreactivity, respectively.

To determine whether tryptase and CitH3 were present in methadone-induced MCETosis, we tested two methadone concentrations and incubation times. Control cells treated with vehicle (VEH) showed cytoplasmic tryptase and intranuclear CitH3 (Figure 3). Methadone (0.5 mM) induced the clustering of MCs after a 2.5 min exposure and some tryptase-containing DNA traps were evident after 5 min. Exposure to 1 mM methadone for 2.5 min resulted in MCs clustering, nuclear content extrusion, and formation of ET positive to tryptase and CitH3 (white and yellow arrows). The same methadone concentration induced the rupture of all cells after 5 min of incubation. A 4 h PMA treatment caused effects comparable to those produced by 0.5 mM methadone for 5 min (Supplementary Materials Figure S2).

In the remaining experiments, we used BMMCs treated with 0.5 mM methadone for 5 min to identify the receptors and signaling pathways involved in methadone-induced MCET formation.

### 2.4. ROS Production and Intracellular Ca^2+^ Increase Are Critical for Methadone-Induced Cell Death in BMMCs

**Figure 4:**
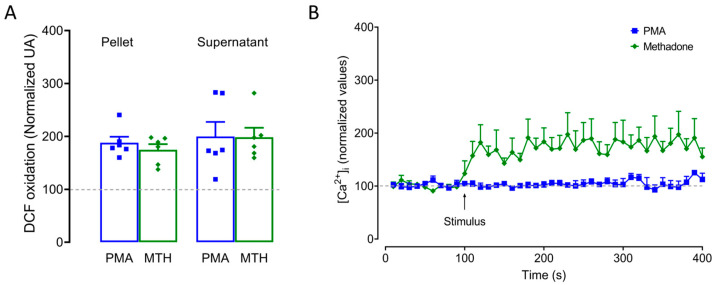
Methadone triggers ROS production and increases [Ca^2+^]*i* in BMMCs. (**A**) Five-hundred thousand cells were loaded with DCF-DA for 30 min and then treated with PMA (1 µM for 4 h) or 0.5 methadone for 5 min. The fluorescence intensity of the pellet and supernatant was used to measure intracellular and extracellular ROS production, respectively, using different BMMC cultures for each condition. Data were normalized to vehicle-treated BMMCs. (**B**) Five million BMMCs were loaded with Fura-2-AM, and basal fluorescence was recorded for 100 s, followed by stimulation with 1 µM PMA or 0.5 mM methadone (MTH) for up to 300 s. For reasons of clarity, values for vehicle-treated cells are not shown.

To analyze whether ROS production was involved in methadone-induced MCETosis, we used DCFH2-DA, a compound that changes to fluorescent 2′,7′-dichlorofluorescein (DCF) in the presence of ROS. PMA and methadone almost doubled ROS levels in cell pellets and supernatants compared to control cells. No differences were observed between PMA and methadone, pellets, or supernatants (F_(3,20)_ = 0.74, *p* = 0.4; one-way ANOVA; Figure 4A). Next, we used the Ca^2+^ indicator Fura 2-AM to analyze the role of intracellular calcium concentration ([Ca^2+^]*i*) in methadone-induced cell death. Methadone produced a maximum [Ca^2+^]*i* increase of 197.6 ± 34.1 nM compared to 125.3 ± 6.1 nM produced by PMA and 123.5 ± 3.5 nM observed in control BMMCs (Figure 4B).

**Figure 5:**
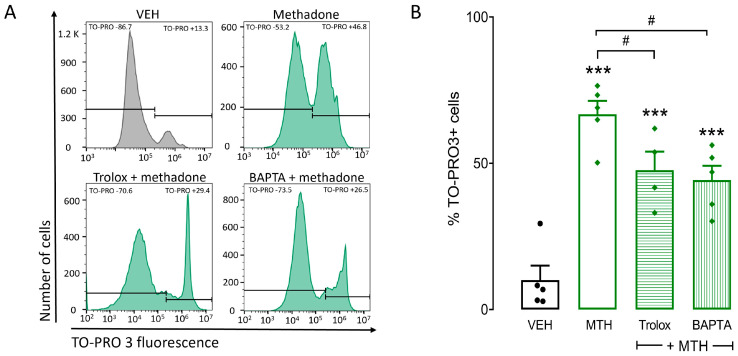
Methadone-induced BMMC death depends on increased [Ca^2+^]*i*. (**A**) One million BMMCs were incubated with vehicle (VEH) Trolox (10 mM) or BAPTA (50 µM) for 15 min before exposure to 0.5 mM methadone for 5 min. Cells were then loaded with TO-PRO3, and cell viability was measured by flow cytometry. (**A**) Representative flow cytometry histograms corresponding to different treatments. Horizontal lines delimit the fluorescence values corresponding to TO-PRO negative and positive cells. (**B**) Percentage of TO-PRO3-positive cells. *** *p* < 0.001 vs. control, # *p* < 0.05 vs. methadone; Dunnett’s test. *n* = 4–5 experiments using different BMMC cultures for each condition.

To determine whether ROS production and Ca^2+^ increase participated in methadone-induced cell death, BMMCs were exposed to the antioxidant Trolox (10 mM) or the calcium chelator BAPTA (50 µM) 15 min before methadone administration. We selected these concentrations because they have been used previously on BMMCs to block the increase in ROS and calcium under distinct stimuli [24]. Trolox and BAPTA pretreatments prevented methadone effects on cell death. Methadone induced 66.7% of cell death, and Trolox and BAPTA diminished that value to 47.6% and 44.2% (F_(2,11)_ = 5.7, *p* < 0.01, one-way ANOVA; Figure 5). This result indicates that ROS production and [Ca^2+^]*i* increase play a significant role in cell death caused by methadone.

### 2.5. μ-Opioid and TLR4 Receptors Participate in Methadone-Induced Cell Death in BMMCs

**Figure 6:**
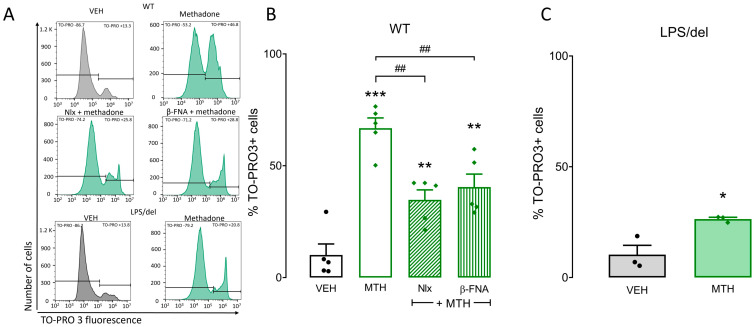
Methadone-induced cell death in BMMCs depends on µ-opioid and TLR4 receptors. One million BMMCs were pretreated with 10 µM naloxone or 1 µM β-FNA for 15 min before exposing them to 0.5 mM methadone (MTH) for 5 min. Cells were then loaded with TO-PRO3 to determine their viability by flow cytometry. (**A**) Representative flow cytometry histograms. Horizontal lines delimit the fluorescence values corresponding to TO-PRO negative and positive cells. (**B**) Percentage of TO-PRO3-positive cells in distinct conditions. ** *p* < 0.01, *** *p* < 0.001 vs. vehicle (VEH); ## *p* < 0.01 vs. methadone; Dunnett’s test. *n* = 5 experiments for each condition using different BMMC cultures. (**C**) Percentage of TO-PRO3-positive cells in distinct conditions. * *p* < 0.05 vs. vehicle; Student’s t test. *n* = 3 experiments for each condition using different BMMC cultures.

To evaluate the role of µ-opioid receptors in methadone-induced cell death, we pretreated BMMCs with 10 µM of the non-selective opioid receptor antagonist naloxone or 1 µM of the selective, irreversible μ-opioid receptor antagonist β-FNA for 15 min before methadone addition. Naloxone decreased methadone-induced cell death from 66.7% to 34.7%, whereas β-FNA reduced it to 40.4% (F_(2,12)_ = 11.75, *p* < 0.001, one-way ANOVA; Figure 6B). To analyze the role of TLR4 receptors, we generated BMMCs from LPS/del mice, a strain that does not express these receptors. Methadone produced 26.4% cell death in TLR4-defective BMMCs (Figure 6C), i.e., 40% less than in WT BMMCs subjected to the same treatment (t = 3.75; *p* = 0.01, Student’s *t*-test). These experiments show that methadone produces death of BMMCs, acting on both the µ-opioid and TLR4 receptors.

### 2.6. µ-Opioid Receptors Require TLR4 Receptors to Mediate Methadone-Induced ROS Production

**Figure 7:**
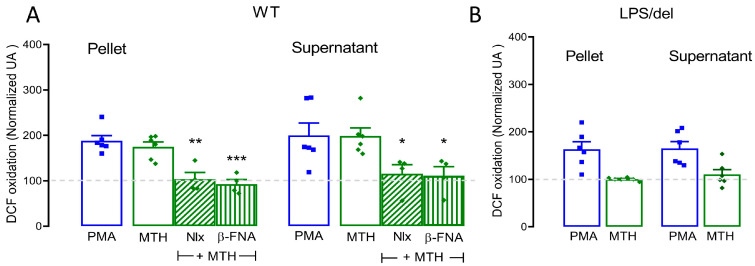
Methadone-induced ROS production is dependent on µ-opioid and TLR4 receptors. (**A**) Five-hundred thousand cells were loaded with DCF-DA for 30 min, then pretreated with naloxone (10 µM) or β-FNA (1 µM) for 15 min before being exposed to 0.5 mM methadone (MTH) for 5 min. The fluorescence intensity of the pellet and supernatant was used to measure intracellular and extracellular ROS production, respectively; * *p* < 0.05, ** *p* < 0.01, *** *p* < 0.001 vs. methadone, one-way ANOVA. *n* = 4–7 experiments for each condition using different BMMC cultures. (**B**) Same treatment as in A, using LPS/del BMMCs. *n* = 6 experiments for each condition using different BMMC cultures. All data were normalized to values obtained from vehicle-treated BMMCs (dotted line).

To determine whether µ-opioid receptors or TLR4 receptors participate in methadone-mediated ROS production, we treated BMMCs for 15 min with 10 µM naloxone or 1 µM β-FNA before methadone and measured ROS production 5 min later. Both opioid receptor antagonists decreased methadone-induced ROS levels (pellet: F_(2,11)_ = 15.59, *p* < 0.001; supernatant: F_(2,11)_ = 7.134, *p* < 0.05, one-way ANOVA; Figure 7A). There were no significant differences between the ROS produced by PMA or 0.5 mM methadone in the pellet or supernatant (pellet: F_(3,16)_ = 16.5, *p* < 0.72; supernatant: F_(3,16)_ = 4.525, *p* ˃ 0.9, one-way ANOVA). We also measured ROS production in BMMCs derived from LPS/del mice stimulated with methadone. Methadone failed to produce ROS in these TLR4-defective BMMCs. Together, those results showed that µ-opioid receptors are necessary for methadone-induced ROS production, but they require the presence of TLR4 receptor to mediate that effect.

### 2.7. µ-Opioid Receptors Induce Calcium Mobilization in BMMCs

**Figure 8:**
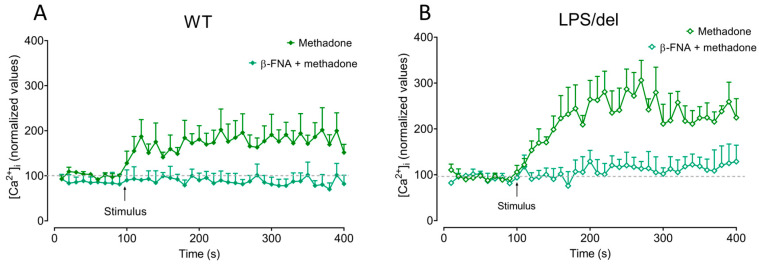
The increase in [Ca^2+^]*i* produced by methadone depends on the µ-opioid receptor. (**A**) Five million WT BMMCs were loaded with Fura-2-AM, pretreated or not with 1 µM β-FNA for 15 min. Basal fluorescence was recorded for 100 s. Then, methadone (0.5 mM final concentration) was added, and the response recorded for additional 300 s. (**B**) Calcium trace of TLR4-defective BMMCs treated with methadone or β-FNA plus methadone. *n* = 3–6 experiments for each condition using different BMMC cultures.

To investigate whether µ-opioid receptors contributed to methadone-mediated [Ca^2+^]*i* increase, cells were treated with 1 µM β-FNA for 15 min. To evaluate the role of TLR4, we used BMMCs generated from LPS/del mice. β-FNA treatment diminished methadone-induced [Ca^2+^]*i* increase in WT BMMCs (Figure 8A). Methadone also induced calcium mobilization in LPS/del cells, and this process was sensitive to β-FNA treatment (Figure 8B). These results show that the µ-opioid receptor is responsible for methadone-induced [Ca^2+^]*i* increase in BMMCs.

### 2.8. µ-Opioid and TLR4 Receptors Share Intracellular Localization in BMMCs

**Figure 9:**
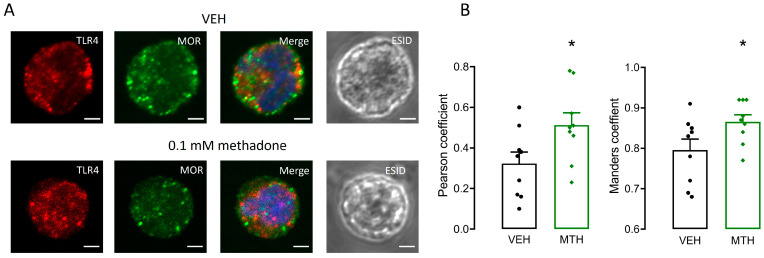
Methadone treatment increases intracellular co-localization of µ-opioid and TLR4 receptors in BMMCs. (**A**) Representative confocal microscopy images of vehicle (VEH)- or methadone (MTH)-treated cells. Red: TLR4 receptor, green: µ-opioid receptors (MOR), blue: DNA. Scale bar: 2 µm, objective: 63×. Images from three distinct experiments performed with independent BMMC cultures. (**B**) Analysis of µ-opioid and TLR4 receptors colocalization by Pearson’s and Manders’ coefficients. * *p* < 0.05 vs. vehicle; Student’s *t*-test. Mean values ± SEM of 9 cells of 3 experiments using different BMMC cultures.

Because the μ-opioid receptor was responsible for methadone-induced ROS production and that effect was not observed in the absence of TLR4 receptors, evidence of possible interaction between those molecules was collected utilizing confocal microscopy. BMMCs were treated with vehicle or 0.1 mM methadone for 10 min before being processed for immunodetection of TLR4 and μ-opioid receptors. In vehicle-treated cells, both receptors were located in intracellular compartments (Figure 9A), showing detectable colocalization (Pearson’s coefficient = 0.32 ± 0.05 and Manders’ coefficient = 0.79 ± 0.02; Figure 9B), which was significantly increased by methadone treatment (Pearson’s coefficient: F_(8,8)_ = 1.170; *p* < 0.05; Manders’ coefficient: F_(8,8)_ = 2.444; *p* < 0.05; Student’s *t*-test; Figure 9B). These results show that part of the µ-opioid and TLR4 receptors are located in the same intracellular compartments and that methadone treatment increases their recruitment to those sites.

## 3. Discussion

Opioids have complex immunomodulatory properties [30,31]. They modify pathogen-induced cytokine secretion, microglial activation, lymphocyte maturation, and activation of macrophages and natural killer cells, among other effects [4,7]. In addition, opioids can induce cell death through different mechanisms, such as apoptosis, pyroptosis, and necrosis [4,7,12,13,26]. The receptors involved and the type of cell death differ according to the opioid compound, administration protocol, and cell type analyzed.

**Figure 10:**
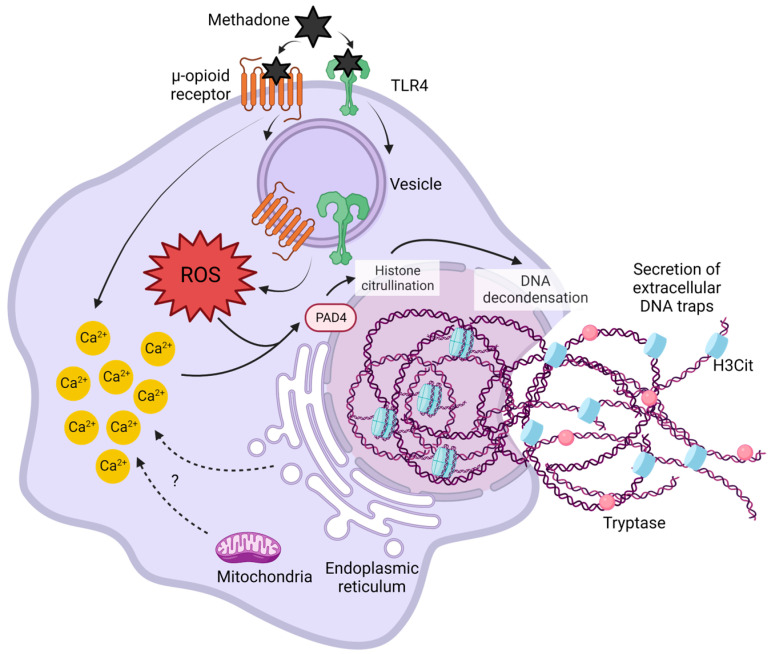
Methadone induces MCETosis by activating µ-opioid and TLR4 receptors. Methadone induces the formation of ETs in BMMCs by a mechanism that depends on ROS production and calcium mobilization. ROS generation after methadone treatment relies on μ-opioid receptor activation but requires the presence of TLR4 receptors, probably through close interaction occurring in intracellular compartments. Sources of intracellular calcium associated with ET formation in other immune cells are shown (mitochondria and endoplasmic reticulum). ROS production and calcium rise lead to PAD4 activation and histone citrullination. Methadone-induced ETs are decorated with citrullinated histones and tryptase.

Opioids act on GPCRs and TLRs [18,32,33], triggering complex signaling pathways that involve signaling crosstalk to modulate cell fate and inflammation. The present results show the following findings: (1) methadone induces BMMC death by a process consistent with MCETosis; (2) methadone promotes ROS production and increases [Ca^2+^]*i*; (3) ROS and increased [Ca^2+^]*i* are needed for methadone-induced MC death; (4) μ-opioid receptors are mainly responsible for methadone-induced ROS production and calcium mobilization; (5) methadone-promoted ROS production, but not calcium rise, depends on the presence of TLR4 receptors; and (6) μ-opioid and TLR4 receptors are colocalized in BMMCs and methadone further promotes colocalization (Figure 10).

Methadone, an opioid with a high affinity for the μ-opioid receptor, is frequently used for long periods by people with cancer pain or opioid use disorders. The effects of methadone on immune cells have been poorly described and they could involve long-term changes in inflammatory reactions or responses against pathogens. For example, the toxic effects of methadone have been shown in cell cultures [14,34,35,36]. As previously mentioned, opioid exposure can lead to cell death. For example, morphine, the prototypic opioid receptor agonist, induces apoptosis in human neurons and fetal microglia through µ-opioid receptor activation [37].

Considering the fact that 1 mM morphine causes detectable effects on distinct signaling pathways in BMMCs [24], we started with 1 mM methadone, going downwards, to evaluate its effects on this cell preparation. Interestingly, 0.5 mM and 1 mM methadone induced the death of 54.17% and 83.2% of cells, respectively. Our results are in line with those reported in leukemia cells, where methadone induced apoptotic cell death at concentrations ranging from low to medium micromolar [13,34,36]. In the present study, we observed cell death at relatively high methadone concentrations (0.5 mM and 1 mM), suggesting that its deleterious effects occur at a wide concentration range.

Our results show that methadone requires the convergence of diverse mechanisms for cell death to occur, particularly μ-opioid and TLR4 receptor activation. However, we cannot rule out the participation of other molecules that could lead to cellular stress and cell death. Methadone activates distinct potassium channels [38,39,40], can act as a calcium ionophore [26] and causes cell death by the impairment of bioenergetic metabolism [13,26]. Whether those actions are involved in the rapid effect observed in BMMCs is still an open question.

Our results show that methadone causes extracellular DNA trap formation-associated cell death in BMMCs (MCETosis). To the best of our knowledge, this is the first report of this type of cell death produced by methadone. Evidence from the literature indicates that µ-opioid receptor activation can induce different types of cell death. For example, methadone increased the cleaved caspase3/pro-caspase3 ratio, suggesting the induction of apoptosis in Sertoli (TM4) cells subjected to an in vitro model of opioid dependence [41]. In another study, chronic methadone treatment increased the expression of necroptosis-associated markers in the cerebellum of rats [42].

ET formation was first described as an early event induced by microbes in neutrophils. Since then, the primary function of ETs has been associated with the rapid killing of bacteria and the potentiation of inflammation [43]. The present results show that methadone induces MCETosis approximately 2.5 min after stimulation (Figure 3). This time is very short compared to that which has been observed with other cells and activators. However, there are also reports indicating that the formation of ETs can occur shortly after stimulation. For example, in HL-60 granulocytes and human neutrophils, TNF-α induces detectable ET formation in 10 to 15 min [43,44]; *Staphylococcus aureus*-induced nuclear envelope breakdown and DNA release can occur as early as 5 min after contact with human neutrophils [45]. In response to pathogens, some cytokines (e.g., IL-1β or IL-23) or chemical compounds like PMA, MCET formation is triggered by activating nicotinamide adenine dinucleotide phosphate (NADPH) oxidase, leading to ROS production and an increase in intracellular calcium [21,22]. Those molecular changes result in peptidylarginine deiminase 4 (PAD4) activation, which catalyzes the citrullination of histones, causing chromatin decondensation and cell death [46].

Our data show that opioid and TLR4 receptors are involved in methadone-induced MCETosis in BMMCs. Receptor-mediated ET formation has been observed in distinct contexts; for example, receptors for advanced glycation products (RAGE), IgG receptors (FcγR), C-type lectin receptors, and other innate-immunity-associated molecules are coupled to signaling cascades leading to MAPK activation, calcium increase, and ROS production that eventually led to the extrusion of DNA in neutrophils and other cells [47,48]. Also, dopaminergic receptors have been proposed to mediate the formation of dopamine-induced ETs in glioblastoma cells [49], suggesting that stimulation of GPCRs other than opioids can also lead to ETosis.

As shown in the present study, μ-opioid receptor activation contributes to methadone-induced cell death, ROS production, and calcium mobilization, whereas TLR4 receptors are required for μ-opioid receptor-mediated ROS production but not for calcium mobilization. The main mechanism proposed for opioid-dependent intracellular calcium rise in immune cells is activating the β-isoform of phospholipase C (PLC) by the βγ subunits of G proteins [13,50]. However, although GPCR activation can lead to ROS production [51], our results strongly suggest that μ-opioid receptor-mediated ROS generation induced by methadone requires the presence of TLR4 receptors.

Actions of opioids in immune cells have been generally described as immunosuppressive through activation of μ-opioid receptors [9,52], although other classical opioid receptors (such as the δ subtype) have also been involved [53]. On the other hand, the proinflammatory actions of opioids in immune cells (e.g., microglia) have been associated with the interaction of these compounds with TLR4 receptors [18,54,55]. Evidence of opioid interaction with opioid and TLR4 receptors has led to the hypothesis that some in vivo actions of opiates are mediated by their proinflammatory actions through TLR4 triggering, whereas other effects are more related to the direct activation of classical GPCR-dependent signaling pathways [18,33,55,56]. Our data suggest that, for the induction of MCETosis, methadone activates μ-opioid receptors, but part of the signaling machinery needed to induce cell death relies on TLR4 receptor activation.

Distinct mechanisms of crosstalk between opioid and TLR receptors have been described. For example, µ-opioid signaling can interrupt TLR4 activation [24,31,57], or µ-opioid and TLR4 receptors can work alongside to activate MAPK or improve the release of proinflammatory mediators and cytokines [58,59,60]. The molecular mechanisms behind those pathways are far from being fully elucidated, as they could involve the production of intermediary compounds to coordinate the activation of one receptor after the other. One exciting possibility is that methadone could induce a close molecular interaction between μ-opioid and TLR4 receptors, as colocalization of both proteins was detected by confocal microscopy, and this effect increased after methadone treatment. In other experimental models, direct interaction between GPCRs and TLR4 receptors to activate specific signaling pathways has been reported. For example, the protease-activated (PAR2) receptor forms a complex with TLR4 receptors to activate the NFκB transcription factor in response to synthetic PAR2 agonist peptides [61]. Such close interaction was found to be responsible for vascular contractility in an in vivo model of endotoxemia in rats and on isolated rat aortic rings [62].

Our results provide experimental evidence to propose a new crosstalk mechanism between TLR4 and μ-opioid receptors in MCs and open new possibilities of μ-opioid and TLR4 receptor interactions (besides direct activation of TLR4 and μ-opioid receptors by opioid compounds). Those events should be taken into account to understand the complex responses of immune cells to opiates. Also, the differential formation of signaling complexes containing both TLR4 and μ-opioid receptors in distinct cells could explain, at least in part, the diverse range of opioid actions. Further analysis of that interaction and the mechanism by which methadone promotes it, causing MCETosis, is a matter for future studies.

## 4. Materials and Methods

### 4.1. Animals

We used 20 C57BL/6J (stock no. 000664) and 15 B6.B10ScN-TLR4^LPS/del^JthJ (stock No. 007227) mice from The Jackson Laboratory (Bar Harbor, ME, USA). Animals were kept under controlled temperature (22 to 24 °C) and humidity, with free access to food and water. Experimental procedures were approved by our Institutional Committee for the Care and Use of Laboratory Animals (CICUAL protocols 0137-15; 0074-13) and followed the ARRIVE guidelines for animal research [63].

### 4.2. Drugs, Reagents, and Antibodies

(_D-L_)-Methadone hydrochloride was kindly donated by the National Institute of Drug Abuse (NIDA) Drug Supply Program (North Carolina, United States of America). Naloxone hydrochloride (cat. Number: N-7758), β-funaltrexamine hydrochloride (β-FNA; cat. Number: 72786-10-8), and phorbol 12-myristate 13-acetate (PMA; cat. Number: P-1585) were bought from Sigma Aldrich (St. Louis, MO, USA). Based on previous reports from our lab and others, the methadone concentrations used in this paper ranged from 0.1 to 1 mM [24,26,64]. Opioids were dissolved in a sterile 0.9% saline solution before use. PMA was dissolved in 10% DMSO (cat. Number: D8418). The antioxidant Trolox (cat. Number: 648471; Merck Millipore, Burlington, MA, USA) and the calcium chelator BAPTA (Merck Millipore 2787, Burlington, MA, USA) were obtained from Calbiochem (Darmstadt, Germany).

TO-PRO^TM^-3 iodide (cat. Number: T3605) was acquired from Thermo Fisher Scientific (Waltham, MA, USA) and used as a cell viability marker in a 1:1000 dilution. For immunofluorescence, we used the following antibodies: Anti-histone H3 (citrulline R2 + R8 + R17) (cat. Number: ab2378) was from Abcam (Cambridge, UK); mast cell tryptase (cat. Number: SC-32889) from Santa Cruz Biotechnology (Dallas, TX, USA); anti-mouse CD284 (TLR4) (cat. Number 145401) from BioLegend (San Diego, CA, USA); anti- µ-opioid receptor (cat. Number: MAB8629) from R&D Systems (Minneapolis, MN, USA); donkey anti-rabbit Alexa 568 (cat. Number: A10042) and donkey anti-rabbit Alexa 488 (cat. Number A21206) were from Invitrogen (Waltham, MA, USA); donkey anti-mouse Alexa 647 (cat. Number: A31571) and donkey anti-rat Alexa 594 (cat. Number A21209) were from Life Technologies (Carlsbad, CA, USA); DAPI (cat. Number: D3571) was from Molecular Probes (Eugene, OR, USA). For scanning microscopy, reagents were provided by Laboratorios Nacionales de Servicios Experimentales (LANSE; CINVESTAV; Mexico City, Mexico). 2′7′-dichlorodihydrofluorescein (DCFH_2_-DA; cat. Number 2044-85-1), Fura 2-AM (cat. Number: 108964-32-5), and anti-DNP IgE (cat. Number: D8406) were obtained from Sigma-Aldrich (St. Luis, MO, USA). RPMI 1640 cell culture media (cat. Number: R4130), bovine serum (FBS; (cat. Number: 26140079), penicillin (cat. Number: 15140122), antibiotic/antimycotic mixture (cat. Number: 15240062), pyruvate (cat. Number: 11360070), nonessential amino acids (cat. Number: 11140050), bovine serum albumin (BSA), EDTA-trypsin, and Prolong^TM^ Diamond Antifade mountant (cat. Number P36970) were from Invitrogen (Waltham, MA, USA). TNF-α ELISA kit (cat. Number: 900-K54) and recombinant murine interleukin 3 (IL-3; cat. Number: 213-13x) were from Peprotech (Rocky Hill, NJ, USA).

### 4.3. BMMC Cultures

Bone marrow was isolated from the tibia of 5–6-week-old WT or TLR4^LPS/del^ mice. Bone marrow was cultured in 30 mL of RPMI medium supplemented with heat-inactivated 3 mL fetal bovine serum (FBS), 20 ng/mL IL-3, 20 mM HEPES, 1× of nonessential amino acids, 1 mM pyruvate, 100 UI/mL penicillin, and 10 µg/mL streptomycin. For the TLR4^LPS/del^ cultures medium, 10 ng/mL of stem cell factor was added. The cell cultures were maintained in a humidified 37 °C incubator with 5% CO_2_, changing the medium weekly. After 5–6 weeks, BMMCs were monitored by flow cytometry using a specific antibody against the FcεRI α-chain (cat. Number: 17-5898-80) from Life Biosciences (Boston, MA, USA), and only cultures with at least 98% of cells positive for this receptor were used. For in vitro experiments, BMMCs were sensitized with 100 ng/mL monoclonal anti-DNP IgE for 18 h.

### 4.4. Viability Test

BMMC viability was assessed after each treatment. Half a million BMMCs were resuspended in 100 µL of PBS 1X with TO-PRO^TM^-3 (1:1000 dilution). Cells were incubated for 5 min at room temperature and then analyzed by flow cytometry (Cytoflex S, Beckman Coulter, Brea, CA, USA). Sample analysis was performed using the software FlowJ 10.8.1 (Ashlan, OR, USA).

### 4.5. Immunofluorescence

To observe ETs, 0.5 × 10^6^ BMMCs were resuspended in 10 µL PBS 1X with DAPI (1:500 dilution), after which, the cells were placed in electrically charged slides for 10 min. To determine tryptase and CitH3 in MCETs, 0.5 × 10^6^ BMMCs were put in loaded slides for 10 min. After that, they were washed once with PBS 1X; ice-cold 4% PFA for fixation was added for 20 min. Blocking was performed for 1 h using a solution of 4% BSA, 5% donkey serum, and 0.05% tween 20. After blocking at room temperature, cells were incubated with the specific primary Abs (rat anti-Histone H3 and rabbit anti-mast cell tryptase) and diluted in blocking solution overnight at 4 °C. Dilutions of both primary Abs were 1:300. Then, ten distinct washed with PBS 1X were performed, followed by incubation with secondary Abs (donkey anti-rabbit Alexa 568 and donkey anti-mouse Alexa 647; 1:400) and DAPI (1:500 dilution) for 2 h. Slides were then washed with PBS 1X. Slides were covered with Antifade Reagent and sealed using a coverslip. To analyze µ-opioid and TLR4 receptors intracellular localization, 0.5 × 10^6^ BMMCs were subjected to the same process described for MCETs with the following antibodies: primary Abs rabbit anti-µ-opioid receptor (1:350) and rat anti-TLR4 receptor (1:300), and secondary abs donkey anti-rat 594 (1:400) and donkey anti-rabbit 488 (1:500). Cells were analyzed by confocal microscopy using a Zeiss LSM 800 model AX10 (Oberkochen, Germany) and the ZEN 2.3 blue edition software. Manders and Pearson coefficients were calculated utilizing the ZEN2.3 blue edition.

### 4.6. Scanning Electron Microscopy

One million BMMCs were left to stand in electrically charged slides for 10 min. Then, cells were fixed with 2.5% glutaraldehyde for 1 h at room temperature. After fixing, samples were washed thrice with PBS 1X and post-fixed with 1% osmium tetroxide (OsO_4_) for 1 h. Slides were washed three times with PBS 1X and dehydrated in graded ethanol dilutions: first 50% ethanol, followed by 60% ethanol, and successively by 70%, 80%, 90%, and 100% ethanol, for 10 min each dilution. Finally, samples were dried in a chamber of critical drying point and were coated with a gold–argon method. We used a JSM-6510LV scanning electron microscope JEOL (Tokyo, Japan) to scan the cells.

### 4.7. Determination of ROS Production

Intracellular ROS production was determined utilizing the fluorochrome 2′7′-Dichlorofluorescein diacetate (DCFH_2_-DA). Briefly, 0.5 × 106 BMMCs were incubated with 10 µM DCFH_2_-DA in Tyrode’s buffer for 30 min at 37 °C. Then, cells were centrifuged at 750× *g* for 5 min at room temperature; the supernatant was discarded, and cells were re-suspended in 500 µL Tyrode’s buffer. Cells were stimulated with the vehicle, 0.5 mM Methadone or PMA (25 nM concentration). At the end of the stimulus, cells were centrifuged at 12,000× *g* for 5 min at 4 °C. The supernatant was recollected, and the cell pellets were disrupted by 300 µL of IGEPAL (0.1%) and pipetted vigorously. Subsequently, samples (pellet and supernatant) were centrifuged at 12,000× *g* for 5 min. A measure of 200 µL of the samples were placed in a 96-well black plate. Finally, the fluorescence of each sample was measured on a BioTek Microplate luminometer (FLx800) from BioteK (Winooski, VT, USA) with λ_excitation/emission_ = 488/565. Intracellular and extracellular ROS are shown as arbitrary fluorescence units concerning the basal vehicle stimulation.

### 4.8. Determination of Intracellular Calcium

Intracellular Ca^2+^ concentration ([Ca^2+^]*i*) was determined with Fura 2-AM. Five million BMMCs were loaded with 1 µM Fura 2-AM for 15 min at 37 °C. After that, cells were washed two times, re-suspended in 1 mL of Tyrode’s/BSA buffer, and placed in a cuvette. The cells’ changes in fluorescence were determined in a Fluoromax x spectrofluorometer (Jobin Yvon, Horiba, Kyoto, Japan) a λ_excitation/emission_ = 340/510 nm in intervals of 1.16 s. The cells were held at 37 °C and under agitation during the experiment. Cells were recorded for 100 s (basal fluorescence) and, after 100 s, they were stimulated with PMA or Methadone to the 500 s. The maximum fluorescence (F*_max_*) of each sample was determined by the cells lysing with 10% Triton, and the minimum fluorescence (F*_min_*) was obtained with a Ca^2+^ chelating agent, EGTA (200 nM). Ultimately, the [Ca^2+^]*i* was calculated using the method of Grynkiewicz [65] as follows, where K*d* is the dissociation constant of Fura 2-AM (224 nM):

$$\left[{Ca}^{2+}\right]i=Kd\left(\frac{F-F_{min}}{F_{max}-F}\right)$$

The data were normalized and reported as the fold-change of the [Ca^2+^]*i* basal fluorescence.

## Data Availability

Data are available upon request to the corresponding author.

## Supplementary Materials

The following supporting information can be downloaded at https://www.mdpi.com/article/10.3390/ijms25042137/s1.
