# Supplementary material for: Methadone Requires the Co-Activation of μ-Opioid and Toll-Like-4 Receptors to Produce Extracellular DNA Traps in Bone-Marrow-Derived Mast Cells
Source: Int J Mol Sci. 2024 Feb 10;25(4):2137. doi: 10.3390/ijms25042137 (PMC10889600; doi:10.3390/ijms25042137)
Supplement: Supplementary file 1 [file ijms-25-02137-s001.zip › ijms-2856014-supplementary.pdf]

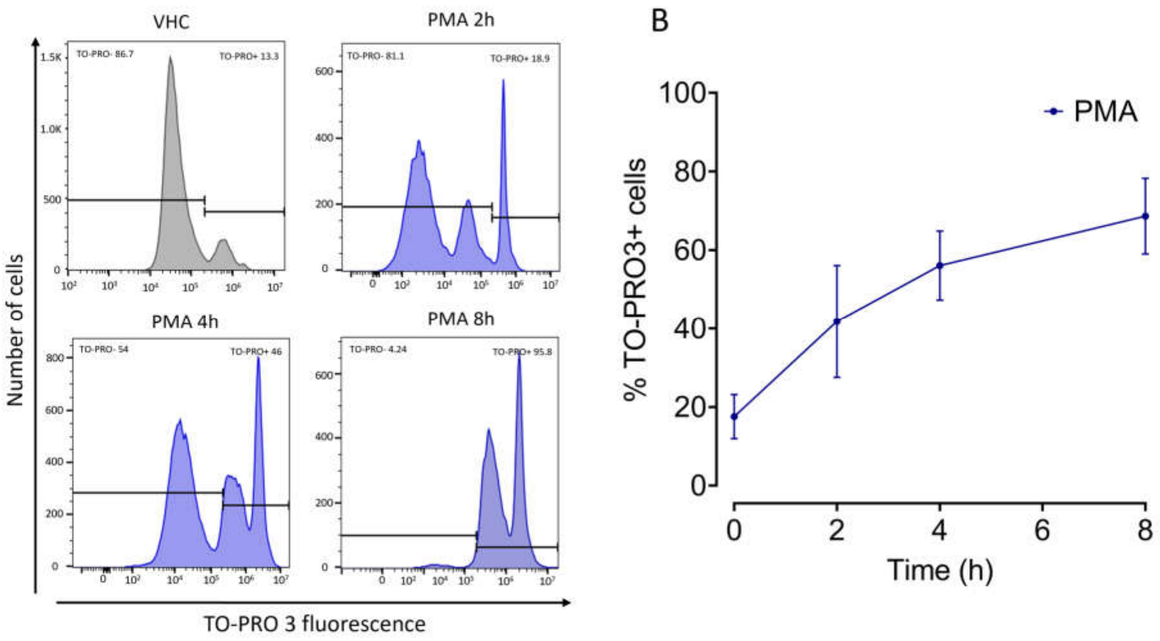

Figure S1. PMA induces BMMC death in a time-dependent manner.

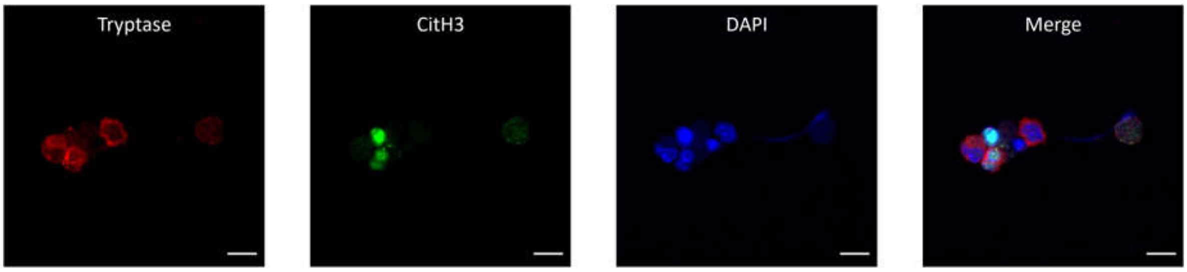

Figure S2. PMA-induced ETs contain Tryptase and CitH3.
